# Factors associated with costs of care in community-dwelling persons with dementia from a third party payer and societal perspective: a cross-sectional study

**DOI:** 10.1186/s12877-020-1414-6

**Published:** 2020-01-16

**Authors:** S. Vandepitte, L. Van Wilder, K. Putman, N. Van Den Noortgate, S. Verhaeghe, J. Trybou, L. Annemans

**Affiliations:** 1 Department of Public Health and Primary Care, Ghent University, Ghent, Belgium; 2 Faculty of Medicine and Pharmacy, Department of Medical Sociology, Vrije Universiteit Brussel, Brussels, Belgium; 3 Department of Internal medicine and Pediatrics, Ghent University, Ghent, Belgium

**Keywords:** Healthcare costs, Resource use, Dementia, Community-dwelling

## Abstract

**Background:**

Besides the importance of estimating the global economic impact of care for persons with dementia, there is an emerging need to identify the key factors associated with this cost. The aim of this study was to analyze associations between the cost of care in community-dwelling persons with dementia and caregiver characteristics from both the healthcare third party payer perspective and the societal perspective.

**Methods:**

Several characteristics based on the cross-sectional data of 355 dyads of informal caregivers and persons with dementia living in Belgium were identified to include in a log-gamma generalized linear model and were used in a multiple linear regression model with bootstrapping to test robustness.

**Results:**

The mean monthly cost of care for a community-dwelling person with dementia was estimated at € 2339 (95% CI € 2133 – € 2545) per person from a societal perspective and at € 968 (95% CI € 825 – € 1111) per person from a third party payer viewpoint. Informal care accounted for the majority of the monthly costs from the societal perspective. Community based healthcare resource use represented the largest cost from the third party perspective. According to the regression analyses, a higher level of functional dependency of the person with dementia and a higher educational level of the caregiver were associated with a higher monthly cost from both a third party payer perspective and a societal perspective. In addition, being retired and a higher quality of life in the caregivers were associated with a lower monthly cost of care from the societal perspective.

**Conclusions:**

Several characteristics of the caregiver and the person with dementia were associated with the monthly costs of care from a third party payer and a societal perspective. Despite the lack of clear causal relationships, the results of this study can assist policy makers in planning and financing future dementia care.

**Trial registration:**

Clinicaltrials.gov NCT02630446, December 15, 2015.

## Background

Dementia is the most common progressive neurodegenerative disease causing substantial emotional, physical, and financial burden not only for the persons with dementia and their informal caregivers, but also for the society [1, 2]. In 2016, Alzheimer’s Disease International estimated the worldwide economic cost of dementia to be 818 billion dollars and expected an increase towards a trillion dollars by 2018 [3]. Not surprisingly there is a growing concern about the future costs of care for persons with dementia. The absence of an adequate cure, the number of affected people that is expected to double or even triple over the next decades [4], and the fact that healthcare policies are confronted with increasing budget constraints [5] feed these concerns. As a result, several cost of illness studies (focusing on community-dwelling and/or institutionalized persons with dementia) have been published. Typically, the estimated total monthly costs differed largely between studies [2, 5–12], which can partially be explained by differences in healthcare systems between countries, but mainly by the methodological choices in the different studies [2, 13, 14]: the type and amount of included resources, the applied perspective (only a healthcare perspective or a full societal perspective), and the valuation technique to monetize informal care (e.g. opportunity cost method, proxy good method) [15]. Especially the latter can lead to large differences in total estimated costs because, when included, this cost category clearly dominated earlier cost of care studies in dementia [5, 8, 9, 11]. This also underpins the importance of a broad societal perspective because excluding informal care as a cost category would highly underestimate the real economic impact of dementia on the society [14].

Besides the importance of estimating the economic impact of caring for persons with dementia patients, especially for those living at home, there is also an emerging need to identify associations between these costs and several characteristics of persons with dementia and caregivers. In this way, policy makers can be assisted in financing and planning future dementia care. Several studies consistently concluded that total costs increased substantially with rising physical dependence and disease severity [6, 9, 10, 12, 16]. One study found that formal costs of care were higher when the person with dementia and the caregiver did not cohabit while informal care costs decreased when the caregiver was employed [16]. Insights in the association between cost of care and other dyad characteristics (such as burden to the caregiver, coping with behavioral problems, and quality of life of the caregiver) remain however limited and inconclusive [5]. The existing studies moreover did not always strictly focus on dementia, nor on the community setting, did not include all relevant resource use categories, or only calculated direct medical costs. A recent study on the determinants of the societal cost of Alzheimer’s disease [5] identified some consistently significant associated factors in three countries (France, Germany, and the UK), as well as some factors that significantly influenced societal cost in one country, but not in the others. These findings highlight the importance of further collecting evidence in other countries, such as Belgium, to enlarge evidence about both universal as well as local factors associated with the cost of care.

Consequently, evidence into the factors associated with cost of care of community-dwelling dementia patients should be leveled up for efficient allocation of resources and to identify opportunities for planning and cost reductions in dementia care. Also, stronger evidence into these factors from both the third party payer and a societal perspective is needed to allow more comprehensive comparisons between countries and over several healthcare systems. Therefore, the main purpose of this study is to identify the factors associated with the total monthly costs of care in community-dwelling persons with dementia in Belgium from both a healthcare third party payer and a societal perspective.

## Methods

The baseline data from a prospective study titled ‘Effectiveness and cost-effectiveness of an in-home respite care program in supporting informal caregivers of people with dementia’ were used [17, 18]. Detailed information on the study aim and methodology can be consulted in the protocol paper by Vandepitte et al. (2016) [19]. Ethical approval for this study was obtained and the project is registered on clinicaltrials.gov, ID: NCT02630446.

### Participants

A total of 355 dyads of informal caregivers and community-dwelling persons diagnosed with dementia participated in the study. They were broadly recruited through differential sites located in Belgium: six different memory clinics, 16 general practitioners, a geriatric daycare clinic, local info points and expert centers for dementia, several Alzheimer cafés (monthly meeting place for peers), the Flemish Alzheimer league, a Belgian in-home respite care service, a sickness fund, and an independent home healthcare service.

To be eligible for inclusion, the informal caregivers had to provide informal care (ADL, IADL or Supervision tasks), and were not allowed to be serving as a professional healthcare worker in the caregiving role under investigation. Second, the informal caregivers had to be the primary caregiver which implies that they were the main person responsible for the informal care. Third, they had to at least fluently understand Dutch or French. Fourth, informal caregivers suffering from severe cognitive impairment or psychiatric comorbidity were not eligible for inclusion. Finally, the care-recipient had to reside in the community and be officially diagnosed with dementia (Alzheimer disease, vascular dementia, frontotemporal dementia, and lewy body dementia).

### Assessments

Candidate variables for analyzing the association with the cost of care were obtained from existing literature. The following sociodemographic variables were selected for inclusion in the analysis: number of caregivers per person with dementia, age and gender of both dyad members (i.e. person with dementia and his principal informal caregiver), educational level and professional status of the caregiver, and cohabitation and type of relationship within the dyad. In line with previous research [18], educational level was divided in three groups according to the International Standard Classification of Education (ISCED) [20]: low (from early childhood education to lower secondary education), moderate (upper secondary education to post-secondary non-tertiary education), and high level education (short-cycle tertiary education to doctoral degree or equivalent). Caregiver professional status contained the following categories: active on the labor market, currently inactive (due to for example: sick leave, time credit), or being retired. The relationship between the dyad members was divided into: spouse/partner, child, or other family member/friend. Cohabitation was a dichotomous variable.

Additional variables related to the person with dementia were dementia severity, level of dependency, and frequency and impact of behavioral problems***.*** Severity of dementia was measured with the Global Deterioration Scale (GDS) [21], a valid and reliable instrument that distinguishes seven stages of dementia based on cognitive decline. Because all persons with dementia in this study were formally diagnosed with dementia, only the stages 4 until 7 were possible, namely: moderate cognitive decline (stage 4), moderately severe cognitive decline (stage 5), severe cognitive decline (stage 6) and very severe cognitive decline (stage 7). The degree of physical dependency of the person with dementia was collected with the Belgian version of the Katz Index of Independence in ADL [22] which measures a person’s ability to perform Activities of Daily Living (ADL) based on the sum of the scores (8 to 32) on six ADL functions (ranging from 1 to 4, 1 = completely independent, 4 = completely dependent): bathing, dressing, toileting, transferring, continence, and feeding. Second, the degree of disorientation in place and time is also collected with this questionnaire. Finally, the Revised Memory and Behavior Problems Checklist (RMBPC) was used to measure frequency of the problematic behaviors of the persons with dementia and the caregiver reaction to these problems [23]. This validated self-report measure consists of 24 items and three domains (depression, memory-related problems and disruptive behaviors). Two five-point Likert scales are applied, one measuring the frequency of problematic behaviors (ranging from ‘never occurs’ to ‘occurs daily or more often’) and one measuring the extent to which the caregiver is affected by these problematic behaviors (ranging from ‘not at all’ to ‘extremely’).

At the caregiver level, burden, quality of life, and intention to institutionalize the person with dementia were identified as candidate variables for analysis. Burden was obtained with the Zarit Burden Interview (ZBI), a validated self-report questionnaire that examines subjective burden of informal caregivers. The 22-item instrument consists of a five point Likert scale ranging from ‘never’ (0) to ‘nearly always present’ (4) which produces a total score between zero (no burden) to 88 (high burden) [24]. Information on the caregiver’s health-related quality of life and self-perceived health was measured using the EQ-5D-5 L, known as a standardized non-disease specific instrument containing five health-related domains (mobility, self-care, daily activities, pain, and depression/anxiety) to evaluate self-perceived health status [25]. Each domain consists of five categories: no problems, slight problems, moderate problems, severe problems, and extreme problems resulting in a five digit health profile that can be translated with the use of a published algorithm into a utility value ranging between − 0.285 and 1 [26]. The intention to institutionalize the person with dementia was measured with the Desire to Institutionalize Scale [27] that includes six questions evaluating the caregivers’ intention to institutionalize the person with dementia in the last 6 months. The scale is scored dichotomously (‘yes’ or ‘no’) whereby the higher the total score (maximum score = 6), the greater the intention is [19].

Healthcare resource use of persons with dementia was collected with a part of the RUD questionnaire [28] allowing to calculate the total cost of care from either a third party payer or societal perspective. Time spent in caregiving was also measured using a part of the RUD questionnaire [28] in which the caregiver’s time is evaluated by asking how much time is spent on activities of daily living (ADL), instrumental activities of daily living (IADL), and supervision on an average caregiving day in the past 30 days. According to common practice [11], the total reported time was capped at maximum 18 h per day to avoid overestimation [21].

The following cost categories were applied: residential healthcare resource use (e.g. hospitalizations, emergency room consults), community based healthcare resource use (e.g. home help services, outpatient visits, and accommodation), informal healthcare resource use (e.g. time spent in ADL, IADL, and supervision), and non-healthcare resources use (e.g. cleaning service, home repair service, transportation service, meal delivery service, and social worker visits). Detailed information on the different services covered by these categories can be found in Table 1.

**Table 1:** Unit costs and total mean monthly costs per capita

Resource	Unit	Unit cost (€) (3rd PP*/pt co-payment**)	Total mean monthly cost per capita (3rd PP*/pt co-payment**)	Total mean monthly cost *Range*	User (%)^f^
Residential healthcare resources					
Hospitalization	Per 24 h	€ 690 (663/27)	€ 330 (317/13)	€ 0–11,851	74 (21%)
Emergency room consults	Per 24 h	€ 41 (21/20)	€ 0.8 (0.4/0.4)	€ 0–21	34 (9.6%)
Community based healthcare resources					
Outpatient visits					
General practitioner	Per visit	€ 25 (21/4)	€ 25 (21/4)	€ 0–127	241 (67.9%)
Geriatrician	Per visit	€ 40 (28/12)	€ 3 (2/1)	€ 0–40	24 (6.8%)
Neurologist	Per visit	€ 58 (46/12)	€ 17 (14/4)	€ 0–232	101 (28.5%)
Psychiatrist	Per visit	€ 48 (36/12)	€ 2 (2/1)	€ 0–191	9 (2.5%)
Physiotherapist	Per visit	€ 22 (16/6)	€ 54 (40/14)	€ 0–1336	84 (23.7%)
Occupational therapist	Per visit	€ 50 (38/13)	€ 8 (6/2)	€ 0–804	19 (5.4%)
Psychologist	Per visit	€ 51 (0/51)	€ 4 (0/4)	€ 0–202	21 (5.9%)
Other outpatient visits	Per visit	€ 38 (27/10)	€ 10 (0/10)	€ 0–451	50 (14.1%)
Home help services					
Nurse visits	Per service	€ 9 (7/2)	€ 160 (120/40)	€ 0–1138	158 (44.5%)
Home help visits	Per hour	€ 39 (33/6)	€ 296 (250/46)	€ 0–9346	87 (24.5%)
Day sitting service	Per hour	€ 5 (3/2)	€ 48 (33/15)	€ 0–3460	46 (13%)
Night-time care	Per hour	€ 5 (3/2)	€ 2 (2/1)	€ 0–144	12 (3.4%)
In-home respite care	Per 24 h	€ 450 (^c^/65)	€ 1 (0/1)	€ 0–525	1 (0.3%)
Accommodation					
Day care	Per visit/day	€ 64 (50/13)	€ 179 (141/38)	€ 0–1592	99 (27.9%)
Host family respite care	Per 24 h	€ 95 (61/34)	€ 0.4 (0/0)	€ 0–142	1 (0.3%)
Short-stay	Per 24 h	€ 87 (56/31)	€ 33 (21/12)	€ 0–1314	28 (7.9%)
Informal healthcare resources					
ADL tasks					
Employable caregivers^a^	Per hour	€ 21	€ 154^d^	€ 0–3034	80 (22.54%)
Non-employable caregivers^b^	Per hour	€ 7	€ 90^d^	€ 0–1564	131 (36.90%)
IADL tasks					
Employable caregivers^a^	Per hour	€ 21	€ 464^d^	€ 0–5601	137 (38.59%)
Non-employable caregivers^b^	Per hour	€ 7	€ 338.^d^	€ 0–2291	214 (60.28%)
Supervision^e^	Per hour	€ 0	€ 1566^d^	€ 0–10,270	222 (62.5%)
Non-healthcare resources					
Social worker visits	Per hour	€ 14	€ 1^d^	€ 0-42	12 (3.4%)
Cleaning service	Per hour	€ 13	€ 102^d^	€ 0–704	187 (52.7%)
Home repair service	Per hour	€ 12	€ 4^d^	€ 0–462	17 (4.8%)
Meal delivery service	Per visit	€ 6	€ 9^d^	€ 0–169	33 (9.3%)
Transportation service	Per service	€ 4	€ 3^d^	€ 0–214	25 (7%)
Total mean monthly costs 3rd party payer perspective	Per capita		€ 968	€ 0–11,773	
Total mean monthly costs societal perspective	Per capita		€ 2339	€ 66–12,938	

^a^Less than 65 years, ^b^ 65 years and older, ^c^ sponsored by private charity, ^d^ not subsidized by a third party payer, ^e^supervision was set at €0 cost to avoid overestimation, ^f^ user = percentage of total sample who used a certain service, *3rd PP = third party payer, **pt co-payment = patient co-payment

### Cost calculations

Unit costs were obtained via the National Institute for Health and Disability Insurance (NIHDI) using standard fees for regularly insured persons with dementia. Other unit costs were derived from secondary resources like the Agency for Care and Health of the Flemish Government and the Public center for Social Welfare (OCMW/CPAS). Detailed information about the unit cost resources can be found in the Additional file 1. Next, monthly costs of care (obtained in the years 2016 and 2017) were calculated by multiplying the amount of resource use in the month prior to recruitment in the study with the corresponding unit cost (prices of the year 2018) [29]. For some services (hospitalization) resource use was collected over the last 6 months before inclusion. In that case, the collected data were divided by six in order to obtain monthly use. The healthcare third party payer approach only included costs for the government’s healthcare budget (exclusive patient co-payments), while the societal approach included all four identified resource use categories (inclusive patient and caregiver co-payments as well).

The problems of monetizing informal care and the different approaches have been extensively discussed in previous work [15, 30]. In this study, informal care (divided into ADL, IADL, and supervision) was valued using the opportunity cost method which estimates the value of lost informal caregiver benefits due to spending time on providing informal care [15]. Caregivers under the age of 65 were considered to be at productive age and therefore informal care cost was valued at the national average hourly gross wage in Belgium [15, 30, 31]. For caregivers at retirement age (≥65 years), informal care cost was valued at lost leisure time which was, corresponding to previous work, calculated at 35% of the value of lost production [11, 32, 33]. According to similar research, hours spent on supervision were monetized at a zero value cost to avoid overestimation [6, 9, 11].

### Sensitivity analysis

Because valuation of informal care can vary substantially based on the used valuation technique, two additional sensitivity analyses were conducted. In the first scenario, informal care was estimated with the proxy good method. This method values time spent in caregiving at a price of a close market substitute [34]. As such, the hourly gross wage rate of a professional home aide with 5 years of experience in Belgium was used in the analysis (13.73€/h) [35]. In the second scenario, informal care was also valued with the proxy good method, however this time valuing lost leisure time at 100% of the wage rate of a professional home aide instead of 35%. In this way, we tried to deal with the potential downwards bias of falsely assuming that time of retired caregivers is less scarce or less valuable.

### Statistical analysis

#### Descriptive statistics and cost estimation

Continuous variables were represented by means and standard deviations while categorical variables were represented with percentages. Monthly total cost of care per capita as well as monthly total cost of each cost category were calculated and represented as means from both the healthcare third party payer perspective and the societal perspective.

#### Model analysis

The cost data had a right skewed distribution. Therefore a Generalized Linear Model (GLM) was built to investigate the associations of the different candidate factors with cost. A GLM does not require normality or homoscedasticity and allows to make inferences about the mean cost directly [36]. Consequently, this flexible modeling technique avoids difficulties with interpretation and back-transformations, and is therefore often preferred in literature over box-cox and log-transformations [5, 11, 36, 37]. A histogram plot with a fitted distribution curve was used to evaluate the appropriate distribution function [38].

Separate models were developed for each perspective, with ‘total healthcare third party payer cost’ and ‘the societal cost’ as dependent variables. Candidate variables for the models were selected based on unadjusted associations between each independent candidate and the dependent variable. Eligible variables for the model were then simultaneously included in the GLM. Additionally, collinearity diagnostics (Variance Inflation Factor, Tolerance) were performed [39]. Finally, to test robustness of the model, an additional multiple linear regression model with bootstrapping (1000) was calculated [16, 40]. All analyses were conducted in IBM SPSS statistical software (version 24.0).

## Results

### Sample characteristics

The descriptive statistics of the dyads characteristics were already represented in a previous study [18] and again outlined in Table 2.

**Table 2:** Characteristics of the study dyads *N* = 355 & unadjusted associations of each characteristic with monthly cost of care (third party payer & societal perspective

	Percentage (n)	Unadjusted association soc persp^a^	Unadjusted association 3rd pp persp^b^
Caregiver age in years, mean (SD)	67.38 (12.04)	*p* < 0.001*^,^°	*p* = 0.446
Caregiver gender, % (n)		*p* = 0.012*	*p* = 0.711
Female	65.4 (232)		
Region, % (n)			
Flanders	52.4 (186)		
Wallonia	38.6 (137)		
Brussels	9 (32)		
Caregiver professional situation, % (n)		*p* < 0.001*	*p* = 0.649
Active on the labor market	20 (71)		
Non-active on the labor market	12.1 (43)		
Retired	67.9 (241)		
Caregiver educational level, % (n)		*p* = 0.001*	*p* = 0.002
Low	27.9 (99)		
Moderate	29.3 (104)		
High	42.8 (152)		
Type of relationship caregiver/person with dementia, % (n)		*p* = 0.111*	*p* = 0.332
Partner/spouse	66.5 (236)		
Daughter/son	26.2 (93)		
Other family member or friend	7.3 (26)		
Cohabitation with person with dementia, % (n)		*p* = 0.309	*p* = 0.362
Yes	79.7 (283)		
Number of caregivers, mean (SD)	1.5 (0.98)	*p* = 0.093	*p* = 0.02
Caregivers burden, mean (SD)	33 (16.18)	*p* < 0.001*^,^°	*p* = 0.012
EQ-5D index, mean (SD)	0.74 (0.22)	*p* < 0.001*	*p* = 0.014
Intention to institutionalize, mean (SD)	1.7 (1.72)	*p* = 0.016*	*p* = 0.005
Time spent in caregiving hours/day, mean (SD)	7.5 (6.21)		
Person with dementia age in years, mean (SD)	78.7 (8.62)	*p* = 0.569	*p* = 0.281
Person with dementia gender, % (n)		*p* = 0.875	*p* = 0.246
Female	53.5 (190)		
Person with dementia educational level, % (n)			
Low	44.2 (156)		
Moderate	26.6 (94)		
High	29.2 (103)		
Severity of dementia, % (n)		*p* < 0.001*^,^°	*p* < 0.001*^,^°
Moderate cognitive decline	29 (103)		
Moderately severe cognitive decline	39.4 (140)		
Severe cognitive decline	25.9 (92)		
Very severe cognitive decline	5.6 (20)		
Level of dependency, mean (SD)	17.2 (6.43)	*p* < 0.001*	*p* < 0.001
Frequency of behavioral problems/last week, mean (SD)	1.4 (0.53)	*p* < 0.001*	*p* = 0.033
Impact of behavioral problems on the caregiver/last week, mean (SD)	1.5 (0.78)	*p* = 0.151*	*p* = 0.360

*eligible for inclusion in the generalized linear model, ^a^societal perspective, ^b^3rd party payer perspective, °excluded in model due to multicollinearity

Regarding the persons with dementia, mean age was 78.7 (SD = 8.62), and slightly more than half were women (53.5%). About 44.2% were low educated, while 29.2% was highly educated. As measured by the GDS, 29% had moderate cognitive decline (GDS 4), 39.4% had moderately severe cognitive decline (GDS 5), 25.9% had severe cognitive decline (GDS 6), and 5.6% had advanced cognitive decline (GDS 7). The average score on the KATZ scale was 17.2 (SD = 6.43), on the behavioral problems frequency scale of the RMBPC: 1.4 (SD = 0.53), and on the behavioral problems reaction scale of the RMBPC: 1.5 (SD = 0.78).

Regarding the caregivers, the mean age was 67.4 years (SD = 12.04) and two third were women (65.4%). 52.4% of them lived in Flanders, 38.6% in Wallonia, and 9% in Brussels. More than two third were retired (67.9%), 20% was active on the labor market and 12.1% was non-active due to several reasons (e.g. sick leave, time credits). The average number of caregivers was 1.5 (SD = 0.98). Almost half of the caregivers had a high education level (42.8%), whereas 27.9% had a low education level. The majority of caregivers were the spouses or partners of the person with dementia (66.5%), whereas only 26.2% were their son or daughter, and the rest were friends or other family members (7.3%). Most caregivers cohabited with the person with dementia (79.7%). The average score on the Zarit Burden Scale was 33.0 (SD = 16.18). Health-related quality of life (5Q-5D-5 L), expressed as a utility score, was on average 0.74 (SD = 0.22). The average score on the desire-to-institutionalize scale was 1.7 (SD = 1.72). Finally, caregivers spent on average 7.5 (SD = 6.21) hours per day on caregiving.

### Costs of care

The total monthly costs of care per capita is summarized in Table 1. From the third party payer viewpoint, the total mean monthly cost of caring for a community-dwelling person with dementia was estimated at € 968, 95% CI [825€-1111€] and from a societal viewpoint at € 2339, 95% CI [2133€-2545€].

**Fig. 1:**
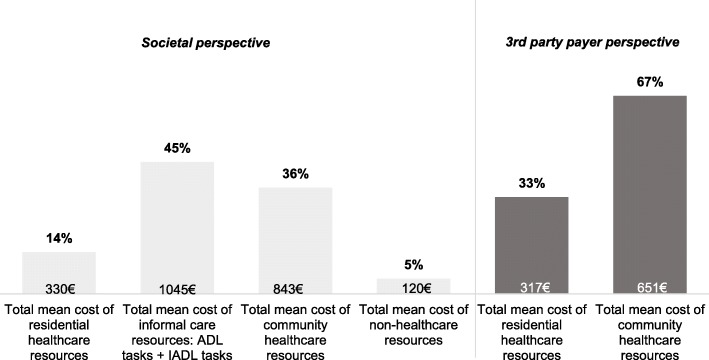
Percentage monthly cost per capita from the societal and third party payer perspective

Figure 1 shows that 67% of the total mean monthly cost from the healthcare third party payer perspective (€ 968; 95% CI [825€-1111€]) was represented by community based healthcare resources (€ 651; 95% CI [559€-743€]), while 33% were attributed to residential healthcare resources (€ 317; 95% CI [204€-431€]). Two major cost categories dominated the total mean monthly societal cost: almost half of the costs could be attributed to informal care resources (45%; € 1045; 95% CI [932€-1159€]), whereas community healthcare resources represented 36% (€ 843; 95% CI [729€-957€]). In the latter category, 60% of costs could be addressed to the subcategory home aid services (€ 507; 95% CI [408€-605€]), 15% to the subcategory outpatient visits (€ 124; 95% CI [107€-141€]), and 25% to the subcategory accommodation (€ 213; 95% CI [170€-256€]). Next, residential healthcare resources and non-healthcare resources only represented 14% (€ 330; 95% CI [212€-448€]) and 5% of the total monthly societal cost (€ 120; 95% CI [105–135]). Finally, the sensitivity analyses showed that informal care costs could vary between 674€ to 1187€ depending on the applied method. Variations in the total cost of care based on these sensitivity analyses are displayed in Table 3.

**Table 3:** Sensitivity analysis of the informal care cost per month based on variations in the valuation of informal care

Societal perspective	Total sample *N* = 355 Mean (95% CI)
Informal care: base case^a^	€ 1045 (€ 0 - € 7205)
Informal care: sensitivity analysis	
Scenario 1^b^	€ 674 (€ 0 - € 4646)
Scenario 2^c^	€ 1187 (€ 0 - € 5046)

^a^Base case: opportunity cost method

^b^Scenario 1: proxy good method, lost leisure time at 35%

^c^Scenario 2: proxy good method, lost leisure time at 100%

### Associations with costs of care: third party payer perspective

Based on the generalized linear model, displayed in Table 4, four dyad characteristics were significantly associated with the total mean monthly cost from a third party payer perspective. The most important association was the level of dependency of the person with dementia (X^2^ = 55.33, Exp (B) = 1.09 [1.07–1.11], *p* < 0.001). One point extra on the KATZ scale (indicating more dependency), was associated with a cost increment of 9%. Quality of life was also a significantly associated factor (X^2^ = 9.62, Exp (B) = 0.38 [0.20–0.70], *p* = 0.002) whereby an increment with 0.1 utility (indicating a better quality of life) was associated with a decrease of monthly costs by 6.2%. The third independently and significantly associated factor was the caregiver desire to institutionalize the person with dementia (X^2^ = 6.15, Exp (B) = 1.1 [1.02–1.19], *p* = 0.013). One point higher on this scale was related with an increase in monthly costs with 10%. Finally, educational level was an important negatively associated factor with monthly costs. The monthly mean costs was 38% lower in the group of caregivers with low education level compared to highly educated caregivers (X^2^ = 9.2, Exp (B) = 0.62 [0.46–0.85], *p* = 0.002). The monthly costs in the group of caregivers with moderate education level were 46% lower as compared to the highly educated caregivers (X^2^ = 15.62, Exp (B) = 0.54 [0.40–0.73], *p* < 0.001).

**Table 4:** Associations with cost of care in community-dwelling persons with dementia: third party payer based on the GLM

	B	Wald	p	EXP (B)	95% Wald Confidence Interval for Exp (B)	
					Lower	Upper
Age of the person with dementia	−0.002	0.048	0.827	0.998	0.982	1.014
Gender of the person with dementia						
[Gender = male]	−0.174	1.530	0.216	0.840	0.637	1.107
[Gender = female]	ref^a^			1		
Amount of caregivers	0.027	0.158	0.691	1.028	0.898	1.176
Educational level caregiver in 3 categories						
[Educational level caregiver = low]	−0.475	9.196	0.002^c*^	0.622	0.457	0.845
[Educational level caregiver = moderate]	−0.618	15.623	0.000*	0.539	0.396	0.732
[Educational level caregiver = high]^a^	ref^a^			1		
Caregiver intention to institutionalize	0.096	6.145	0.013^c^*	1.101	1.020	1.187
Caregiver quality of life - derived from EQ5D	−0.979	9.616	0.002^b^*	0.376	0.202	0.698
Caregiver Burden	0.003	0.262	0.609	1.003	0.992	1.014
Person with dementia level of dependency - KATZ scale	0,085	55.333	0.000*	1.089	1.065	1.113
Frequency of behavioral problems	−0,210	1.991	0.158	0.810	0.605	1.085

^a^reference category, *significant different, ^b^not significant in the linear regression with bootstrapping, ^c^trend towards significance in the linear regression with bootstrapping

When conducting a multiple linear regression with bootstrapping to test robustness of the model, caregiver quality of life was no longer significantly associated with costs of care (*p* = 0.23). Caregiver’s desire to institutionalize the person with dementia only showed a trend towards significance (*p* = 0.13) as well as lower education compared to higher education (*p* = 0.09). In contrast, a moderate level of education remained significantly associated with cost of care compared to a high educational level.

### Associations with costs of care: societal perspective

The adjusted associated factors, as a result of the generalized linear model, are represented in Table 5. From a societal perspective, level of dependency of the person with dementia also represented the most important association (X^2^ = 126.63, Exp (B) = 1.07 [1.06–1.08], *p* < 0.001) with monthly cost of care. When the person with dementia scored one point higher on the KATZ scale (indicating more dependency) costs increased with 7%. Caregiver quality of life was also a significant factor (X^2^ = 8.98, Exp (B) = 0.60 [0.43–0.84], *p* = 0.003). An increase of utility level with 0.1 point was associated with a decrease of monthly costs by 4%. Finally, educational level was also negatively associated with monthly societal cost of care. The monthly mean cost was 18% lower in the group of caregivers with low education compared to high educated caregivers (X^2^ = 4.70, Exp (B) = 0.82 [0.69–0.98], *p* = 0.03). The monthly cost in the group of caregivers with moderate education were 28% lower compared to high educated caregivers (X^2^ = 15.07, Exp (B) = 0.72 [0.61–0.85], *p* < 0.001). In contrast to the third party payer perspective, desire to institutionalize was not significantly associated with societal cost (p=0.229). Two additional characteristics were found to be significantly associated with societal costs: the professional situation and the type of relationship between the caregiver and the person with dementia. More specifically, costs of care increased respectively with 54% and 37% when caregivers were active on the labor market (X^2^ = 11.92, Exp (B) = 1.54 [1.2–1.96], *p* = 0.001) or temporarily inactive (X^2^ = 7.27, Exp (B) = 1.37 [1.09–1.71], *p* = 0.007) compared to retired caregivers. Concerning the relationship between the dyad members, costs decreased with 28% when caregivers were the daughter/son of the person with dementia (X^2^ = 5.03, Exp (B) = 0.72 [0.53–0.96], *p* = 0.025) compared to those who were other family members or friends (read: partner/spouse).

**Table 5:** Associations with cost of care in community-dwelling persons with dementia: societal perspective based on the GLM

	B	Wald	p	EXP (B)	95% Wald Confidence Interval for Exp (B)	
					Lower	Upper
Amount of caregivers	−0.037	0.846	0.358	0.964	0.892	1.042
Gender of the caregiver						
[gender = male]	−0.137	1.807	0.179	0.872	0.714	1.065
[gender = female]	Ref^a^			1		
Gender of the person with dementia						
[gender = male]	−0.015	0.018	0.892	0.985	0.791	1.227
[gender = female]	Ref^a^			1		
Educational level caregiver in 3 categories						
[Educational level caregiver = low]	−0.193	4.695	0.030^b^*	0.824	0.692	0.982
[Educational level caregiver = moderate]	−0.329	15.065	0.000*	0.720	0.610	0.850
[Educational level caregiver = high]^a^	Ref^a^			1		
Professional situation in 3 categories						
[Professional situation = active]	0.429	11.921	0.001*	1.535	1.204	1.958
[Professional situation = nonactive]	0.311	7.270	0.007*	1.365	1.089	1.712
[Professional situation = retired]^a^	Ref^a^			1		
Relationship between caregiver and person with dementia						
[Relationship = partner/spouse]	−0.090	0.294	0.588	0.914	0.659	1.267
[Relationship = daughter/son]	−0.335	5.031	0.025^b^*	0.715	0.533	0.959
[Relationship = other family member/friend]^a^	Ref^a^			1		
Person with dementia level of dependency - KATZ scale	0.069	126.625	0.000*	1.072	1.059	1.085
Age of the person with dementia	−0.002	0.197	0.657	0.998	0.988	1.007
Frequency of behavioral problems	0.060	0.628	0.428	1.062	0.915	1.233
Impact of behavioral problems on caregiver	0.019	0.137	0.712	1.019	0.921	1.127
Caregiver intention to institutionalize	0.023	1.077	0.229	1.023	0.98	1.068
Caregiver quality of life - derived from EQ5D	−0.515	8.980	0.003*	0.597	0.426	0.837

^a^reference category, *significant different, ^b^not significant in the linear regression with bootstrapping

When conducting a multiple linear regression with bootstrapping, the relationship between the person with dementia and his caregiver (*p* = 0.575) was no longer significantly associated with the monthly cost of care. Being a caregiver with low education (compared to high education) only showed a trend towards a significant association (*p* = 0.14). In contrast, a moderate level of education remained significantly associated with cost of care.

## Discussion

This study presents data on the mean monthly costs of care for community-dwelling persons with dementia in Belgium. It also describes the results of a comprehensive analysis into the associations between this monthly cost of care and several person with dementia and caregiver characteristics from a third party payer and a societal perspective. The results from this study can help estimate future costs and effects on the healthcare system, provide data to estimate the impact of supportive strategies and new therapies, and can be incorporated in cost-effectiveness research to assist policy makers in making informed decisions on resource allocation for community-based dementia care.

The average monthly cost of care per capita in community-dwelling persons with dementia was estimated at 968€, (95% CI 825€-1111€) from a third party payer perspective and at a substantially higher cost of 2339€, (95% CI 2133€-2545€) from a societal perspective. This substantially higher cost in the societal perspective can mainly be attributed to the inclusion of informal care, which accounted for 45% of the total societal cost. The latter finding corresponds to earlier studies also concluding that informal care dominated the total costs of care [2, 5, 8, 9, 11]. Community based healthcare resources represent another important cost category. This accounted for 67% of costs in the third party payer perspective and 36% of costs in the societal perspective.

Next, the regression models of both perspectives (societal and third party payer) identified several factors associated with the monthly cost of care in community-dwelling persons with dementia. In accordance to previous studies [9–12, 16], a higher level of dependency was indeed identified as the most import association with higher costs of care. As a result, it seems that the level of dependency is an important universal associated factor. Similar to Dödel et al. (2015) we had to exclude severity of dementia due to its multicollinearity with the level of dependency. This had to be done to avoid problems with model fitting and interpretation of the results. Level of dependency was chosen over severity because it had a stronger association with the costs. Also, the used KATZ scale captures both physical and cognitive dependency, is most used in international research, and is more frequently used in Belgium to value the degree of dependency and support needed. In contrast to previous studies, educational level was identified as another associated factor of monthly cost of care [11, 41]. Being highly educated was associated with higher costs from both perspectives. Although no causality can be assumed this could be explained by several factors. First, it is expected that highly educated people have more financial resources to pay for care and support. Second, highly educated caregivers could be less willing to give up their career to take care for a relative with dementia and therefore call more upon formal services providing care and supervision. Third, they may be better informed about the existence of available formal care services and support. Future research into this association would be valuable in order to investigate if educational level is indeed a universal factor or if its association is more depending on the specific cultural (in terms of: feeling more/less obliged, or willing to care for family) or economic context (in terms of inequality) of a certain county.

Next, from the societal perspective, caregiver quality of life/utility (derived from the EQ5D) was significantly associated with costs of care. Again, without claiming causality, it can be stated that better perceived quality of life was related to lower costs. Current evidence into this mechanism and its direction is scarce. Therefore, it could be interesting to further investigate its relationship as well. Does a higher perceived quality of life (both physical and mental) give caregivers more strength and confidence to deal with the demanding caregiver role and do they therefore utilize less formal care resources? Or do higher monthly costs for care create a lower quality of life due to a high financial burden?

Working status was also significantly associated with societal costs. As such, being retired was associated with lower costs compared to professionally active or temporarily inactive caregivers. Although no causality can be assumed, this association is to be expected since retired caregivers have more time to care for a relative themselves (informal care by retired caregivers costs less than formal care). Another explanation could be that retired caregivers are older and feel more obliged to take care for someone by themselves than younger caregivers. Feelings of guilt when they let someone else take care of a spouse could be more present in this older retired group. Finally, type of relationship between caregiver and person with dementia was associated with the monthly cost of care from a societal perspective only based on the GLM. More specifically, being a child of the dementia person with dementia was significantly associated with less monthly costs of care than being another family member or friend. As expected, the post hoc analyses indicated that caregivers who were another family member or friend used 33% more formal services than caregivers who were a child of the person with dementia. This difference in use of formal care services can indeed explain the difference in monthly societal costs. However, no independent significant difference was found in monthly costs of care for being a partner or spouse compared to being a family member or friend, although persons with dementia of whom the caregiver was the partner/spouse used 69% less formal services. The explanation for this rather counterintuitive result can be found in the fact that spouses/partners provided more than twice as much hours of care per day than others. The latter indeed explains the high monthly cost of care, although most of them were retired and the opportunity cost of informal care hours was valued mostly at the price of lost leisure time.

From the third party payer perspective, the desire to institutionalize was related to the monthly cost of care (only a trend based on the linear model with bootstrapping). Higher desire was associated with higher costs of care. This finding, again not claiming a causal relationship, can be explained by the fact that caregivers with a desire to institutionalize the person with dementia may use more formal services as a ‘rehearsal’ before actual institutionalization or when a crisis occurs while waiting for institutionalization [42, 43]. Also, when caregivers start using services, they become aware of the possibilities [44], barriers are overcome, they accept that they manage everything by themselves, and they even start considering actual placement into a residential setting. Nevertheless, findings must be interpreted with caution since DIS was not significantly associated when evaluating robustness of the model using multiple linear regression with bootstrapping. Further research into this factor and its causal association with costs is thus desirable.

Several important strengths can be addressed to this study. First of all, the sample size of this study is substantially larger than in previous studies [7, 10, 12]. Second, the focus was strictly on informal caregivers of persons with dementia residing in the community while others often only included them as a subsample [7, 45]. This strict focus is preferable, because earlier research indicated that great differences exist in costs of care for community-dwelling persons with dementia compared to institutionalized peers [13]. A third strength is the use of internationally validated tools, as well as the wide range of potentially influencing factors under investigation. Fourth, we used a properly built multivariate model. Additionally, in congruence with previous high quality research, we tested robustness of the model using a linear regression model with bootstrapping [16, 40] As a result, we can be more confident about which factors are related to costs of care. A last important strength of our study is the use of two different perspectives in analyzing the monthly costs and determinants of costs. This makes the cost estimates and conclusions extra valuable for application in planning and financing future dementia care.

Despite the strengths of this study, several limitations should be mentioned and kept in mind when interpreting the results. A first limitation is the cross-sectional nature of this study. Because the data were captured at one point in time, no causality could be assumed and therefore only associations and no predictions could be made. Second, recruitment of the dyads was not at random and was organised in several organizations often specialized in dementia treatment or informal caregiving support. Although not intended nor necessary for the study objectives, it is important to mention that this convenience sample is not representative for the entire dementia caregiver population. For example, caregivers in complete denial, not accepting any help or support, and probably most in need, may not be represented in this study. Also, highly educated caregivers may be overrepresented. Although unambiguous (inter)national numbers of socio-demographic dementia caregiver characteristics are lacking, a recent report of the Flemish government indicated that in general 25.4% of informal caregivers (not dementia specific) is highly educated [46]. This number is lower than the percentage highly educated caregivers in this study (42.8%). Third, although the internationally validated Resource Use in Dementia questionnaire was used to collect cost data, recall bias may be present because caregivers were retrospectively questioned about the time they spent in caregiving on a list of activities during the last month. Also, there might have been an overestimation of caregiving time because several activities can be done at the same time. To deal with this problem, we have excluded supervision from the valuation of informal care time [47]. Fourth, the cost of care for community-dwelling persons with dementia remains subject to high uncertainty due to the lack of a standard methodology to conduct these type of studies [13]. This was confirmed by our sensitivity analyses whereby different valuation methods to estimate informal care costs were executed and proved that informal care costs varied substantially between 674€ and 1187€ based on the used approach. Although it would go beyond the scope of this study, based on the latter finding it could be interesting to investigate what impact these variations in costs of informal care could have on the conducted regression models. Another limitation in the used methodology of valuating the costs of informal care can be found in the fact that we did not have exact wage rates of our participating caregivers. Therefore, we had to use the average wage rate of Belgian employees. However, this could also be considered as a strength because our approach avoids biasing against people with low wage rates. A next limitation that should be discussed is the fact that time spent by retired caregivers is valued at 35% of professionally active caregivers. Although this has been common practice in similar studies [11, 15, 31], this choice remains arbitrary and open for further discussion. However, we have estimated the impact of this arbitrary choice by conducting a sensitivity analysis where time spent by retired caregivers was valued at 100%. Finally, it is important to mention that the cost estimates in this research represent the cost of care in community-dwelling persons with dementia, but that not necessary all costs are caused by the disease itself. Other comorbidities may also have been present and have caused extra financial burden [11, 13].

## Conclusion

Several characteristics of the caregiver and the person with dementia were associated with the monthly costs of care from a third party payer and a societal perspective. Despite the lack of clear causal relationships, the results of this study can assist policy makers in planning and financing future dementia care.

## Supplementary information

**Additional file 1.** Provides detailed information about the unit cost resources.

## Data Availability

The data supporting this article can be made available from the corresponding author on reasonable request.

## Abbreviations

ADL: Activities of daily living
CI: Confidence Interval
EQ-5D-5 L: EuroQol 5 dimensions
GDS: Global deterioration scale
GLM: Generalized Linear Model
IADL: Instrumental Activities of daily living
ISCED: International Standard Classification of Education
NIHDI: National Institute for Health and Disability Insurance
OCMW/CPAS: Public center for Social Welfare
QALY: Quality adjusted life years
RIZIV/INAMI: National Institute for Health and Disability Insurance
RMBPC: Revised memory and behavioral problems checklist
RUD: Resource use in dementia
SD: Standard deviation
ZBI: Zarit burden interview
