# Supplementary material for: Factors associated with costs of care in community-dwelling persons with dementia from a third party payer and societal perspective: a cross-sectional study
Source: BMC Geriatr. 2020 Jan 16;20:18. doi: 10.1186/s12877-020-1414-6 (PMC6966839; doi:10.1186/s12877-020-1414-6)
Supplement: Supplementary file 1 — Additional file 1. Provides detailed information about the unit cost resources. [file 12877_2020_1414_MOESM1_ESM.pdf]

Additional file 1. References resources

Resource	Reference	Reference webpage
Residential healthcare resources		
Hospitalizations (geriatrics, psychiatrics, internal medicine, surgery, neurology)	RIZIV/INAMI	http://www.inami.fgov.be/nl/themas/kost-terugbetaling/door-ziekenfonds/verzorging-ziekenhuizen/Paginas/default.aspx#.Wyu1fWhl-Uk<br>http://www.inami.fgov.be/nl/themas/kost-terugbetaling/door-ziekenfonds/verzorging-ziekenhuizen/Paginas/verpleegdagprijzen-ziekenhuizen.aspx#.Wyu3UWhl-Ul<br>https://www.uzgent.be//nl/patienten/Betaling/Paginas/Factuur.aspx<br>https://tct.fgov.be/webetct/etct-web/html/nl/fbho_faq.jsp<br>https://tct.fgov.be/webetct/etct-web/anonymous?lang=nl
Emergency room consults	RIZIV/INAMI	https://www.cm.be/diensten-en-voordelen/hospitalisatie/verblijf/spoeddienst<br>http://www.riziv.fgov.be/SiteCollectionDocuments/tarief_artsen_deel02_20180101.pdf
Community healthcare resources		
Outpatient visits		
GP/geriatrician/neurologist/psychiatrist	RIZIV/INAMI	http://www.riziv.fgov.be/SiteCollectionDocuments/tarief_artsen_deel01_20180101.pdf
Physiotherapist	RIZIV/INAMI	http://www.riziv.fgov.be/SiteCollectionDocuments/tarief_kinesitherapeuten_20180201.pdf
Occupational therapist	RIZIV/INAMI	http://www.riziv.fgov.be/SiteCollectionDocuments/tarief_revalidatie_verstrekkingen_20180101.pdf
Psychologist	CM	https://www.cm.be/media/PSYCHOTHERAPEUTEN_tcm47-25500.pdf
Home help services		
Nurse visits	RIZIV/INAMI	http://www.riziv.fgov.be/SiteCollectionDocuments/tarief_verpleegkundigen_20180101corr.pdf
Home aid	Flemish Agency for Care and Health	Retrieved from contact person Flemish Agency for Care and Health (department home aid services)
Day sitting service/night-time care	Flemish Agency for Care and Health	https://codex.vlaanderen.be/Zoeken/Document.aspx?DID=1028730&param=inhoud
In-home respite care (Baluchon)	Baluchon Alzheimer Belgium	http://www.baluchon-alzheimer.be/nl/dienstverlening/kost-van-een-baluchonnage.htm
Accommodation		
Day care	Federal government	http://www.ejustice.just.fgov.be/cgi_loi/change_lg.pl?language=nl&la=N&cn=2000062232&table_name=wet
	Partnerplan Probis 2017	http://probis.be/sites/default/files/cases/Presentaties%20PartnerPlan%20Benchmark%202017.pdf
Blankenberge	OCMW	https://www.thuiszorgzakboekje.be/site/zoeken_detail.asp?id=7074&entry_id=69076&rubriekid=16&subrubriekid=13&synoniemid=
Destelbergen	OCMW	http://www.ocmw-destelbergen.be/?q=Dagverzorgingscentrum+De+Triangel
Hasselt	OCMW	https://www.hasselt.be/nl/dagverzorgingscentrum-hogevijf-ocmw-hasselt
Houthalen	OCMW	https://www.houthalen-helchteren.be/dagopvang

Kruibeke	OCMW	https://www.kruibeke.be/product/96/dagopvang-voor-ouderen
Bergen	CPAS	https://www.cpas.mons.be/services/seniors/hebergement-et-soins/bonne-maison-de-bouzanton/services/centre-de-jour
Watermael	CPAS	https://www.centresdesoinsdejour.be/centres-de-soins-de-jour/csj-du-cpas-de-watermael-boitsfort/
Moeskroen	CPAS	https://www.mouscron.be/ma-ville/social/cpas/maisons-de-repos/centre-daccueil-de-jour
Marche	CPAS	https://www.marche.be/social/centre-public-daction-sociale-cpas/centre-daccueil-de-jour/
St.-Agatha-Berchem	CPAS	http://cpasberchem.brussels/sante-et-handicap/le-centre-de-soins-de-jour-le-tournesol-2/
Host family respite care	Flemish Agency for Care and Health	https://codex.vlaanderen.be/Zoeken/Document.aspx?DID=1028549&param=inhoud
		https://www.zorg-en-gezondheid.be/hoeveel-betaalt-een-gebruiker-voor-thuiszorg
Short-stay	Federal government<br>Zorg24 report 2017	http://www.ejustice.just.fgov.be/cgi_loi/change_lg.pl?language=nl&la=N&table_name=wet&cn=2016091607<br>https://online.flippingbook.com/view/585432/118/
Gent	OCMW	http://www.ocmwgent.be/Kortverblijf.html
Brugge	OCMW	https://www.ocmw-brugge.be/kortverblijf#toc-price
Zemst	OCMW	https://www.ocmw-zemst.be/nl/402/content/413/kostprijs.html
Kortrijk	OCMW	https://www.kortrijk.be/zorg/kortverblijf
Merchtem	OCMW	https://www.merchtem.be/product/522/kortverblijf
Charleroi	CPAS	https://www.cpascharleroi.be/index.php/hebergement-aines/centre-de-court-sejour
Bergen	CPAS	https://www.cpas.mons.be/
Bouge	CPAS	https://www.acsol.be/?rub=services#CS
Doornik	CPAS	http://www.cpas-tournai.be/cpas_intranet/index.php?idpage=DET0060102
Erquelinnes	CPAS	http://www.erquelinnes.be/commune/acteurs-locaux/residence-du-quartier-fleuri
Informal care resources		
<u>ADL/IADL tasks</u>		
Employable caregivers (≤65 years)	Salary Survey KULeuven	http://www.vclbleuven.be/wnso/Synthese%20Vacature%20KUL%20salarisenquete%202017.pdf

Non-employable caregivers (≥65 years)	Gustavsson, et al. (2011)	Costs of care in a mild-to-moderate Alzheimer clinical trial sample: key resources and their determinants. Alzheimer's & dementia: the journal of the Alzheimer's Association.
Supervision	Gustavsson et al. (2011)	Costs of care in a mild-to-moderate Alzheimer clinical trial sample: key resources and their determinants. Alzheimer's & dementia: the journal of the Alzheimer's Association.
	Schwarzkopf et al. (2011)	Costs of care for dementia patients in community setting: an analysis for mild and moderate disease stage
	Leicht et al. (2011)	Net costs of dementia by disease stage
Non-healthcare resources		
Social worker visits	OCMW<br>Flemish Government	https://www.vlaanderen.be/nl/vlaamse-overheid/werken-bij-de-vlaamse-overheid/werken-voor-vlaanderen/salarissimulator<br>https://www.jobpunt.be/sites/default/files/vacature/infobrochure/Infobrochure%20maatschappelijk%20werker%20Sociale%20Dienst.pdf
Cleaning service	ACLVB	http://www.aclvb.be/sites/default/files/aclvb/Documenten/Sectoren/loon_arbeidsvoorwaarden/services-aux-entreprises/2018/salaires_cp_121_nl.pdf ; http://www.aclvb.be/nl/pc-32201-loon-en-arbeidsvoorwaarden#lonen
Home repair service		
Zulte	OCMW	http://www.ocmwzulte.be/ocmw/2543-www/2556-www.html
Anzegem	OCMW	http://www.ocmwanzegem.be/website/9-www/51-www.html
Kruishoutem	OCMW	http://www.kruishoutem.be/website/80-www/522-www/102-www/577-www.html?layoutType=print
Wanze	CPAS	http://www.wanze.be/commune/social/cpas/services-accessibles-a-tous/petits%20travaux%20et%20jardinage
Charleroi	CPAS	https://www.cpascharleroi.be/fr/insertion-sociale/pole-d-economie-sociale/proxi-services
Deerlijk	OCMW	http://www.ocmwdeerlijk.be/website/37-www/24-www.html
Dendermonde	OCMW	https://www.dendermonde.be/product/224/klusjesdienst
Meal delivery service		
Berlare	OCMW	https://www.ocmwberlare.be/maaltijden-aan-huis/
Brugge	OCMW	https://www.ocmw-brugge.be/maaltijden-aan-huis
Tienen	OCMW	https://ocmw.tienen.be/maaltijden-aan-huis
Aalst	OCMW	https://www.ocmwaalst.be/page15332317.aspx
Turnhout	OCMW	https://www.ocmwturnhout.be/nl/product_catalog/1737/maaltijden-aan-huis.html-2
Charleroi	CPAS	https://www.cpascharleroi.be/index.php/soins-domicile/repas-domicile
Bergen	CPAS	https://www.cpas.mons.be/services/aide-sociale/aides-a-domicile/repas-a-domicile

Verviers	CPAS	http://www.cpasdeverviers.be/nos-services/services-aines/repas-domicile
Moeskroen	CPAS	https://www.mouscron.be/ma-ville/social/cpas/repas-a-domicile
Andenne	CPAS	https://www.andenne.be/entity/cpas-service-des-repas-chauds-a-domicile/
Transportation service	OCMW - Flemish Health Insurance Fund	https://www.balen.be/product/182/vrijwilligers-gemeente-en-ocmw-minder-mobielen-centrale-aanmelden-vrijwillige-chauffeur https://www.cm.be/diensten-en-voordelen/thuiszorg/vervoer/niet-dringend-ziekenvervoer
